# Glucagon-like peptide-1 receptor agonists for treatment of diabetes and obesity: advantage of oral delivery

**DOI:** 10.3389/fddev.2024.1456654

**Published:** 2024-11-06

**Authors:** R. R. C. New, M. Bogus, G. N. Travers, U. Hahn, A. Vaiceliunaite, M. Burnet, J. H. Wang, H. Wen

**Affiliations:** ^1^ Diabetology Ltd., London, United Kingdom; ^2^ Department of Biomedical Science, Middlesex University, London, United Kingdom; ^3^ Synovo GmbH, Tubingen, Germany; ^4^ Departments of Pharmaceutics (JHW) and Surgery (HW), Xinjiang Medical University, Ürümqi, China

**Keywords:** GLP-1, oral delivery, vagal afferents, diabetes, obesity, peptide

## Abstract

GLP-1 receptor agonists ((GLP-1 RAs) are currently receiving a lot of attention because of their impact in diabetes, weight loss and other areas. While GLP-1 RAs in injectable form are highly efficacious, further work is required to develop oral versions which can deliver these peptides efficiently without requiring use of excessively high doses. This paper describes the ability of an oral peptide delivery formulation, Axcess™, to enhance uptake of GLP-1 receptor agonists via the intestine, resulting in changes in insulin and glucose blood levels indicative of biopotencies of 9% for exendin-4 and 14.8% for semaglutide in preclinical models. The route of delivery suggests that the peptides will be able to interact with the GLP-1 receptors on the vagal afferents of the intestine, as is the case for native GLP-1 in healthy individuals. GLP-1 receptor agonists administered via this route will be a valuable addition to the therapeutic modalities available for treatment of diabetes and obesity.

## 1 Introduction

**FIGURE 1:**
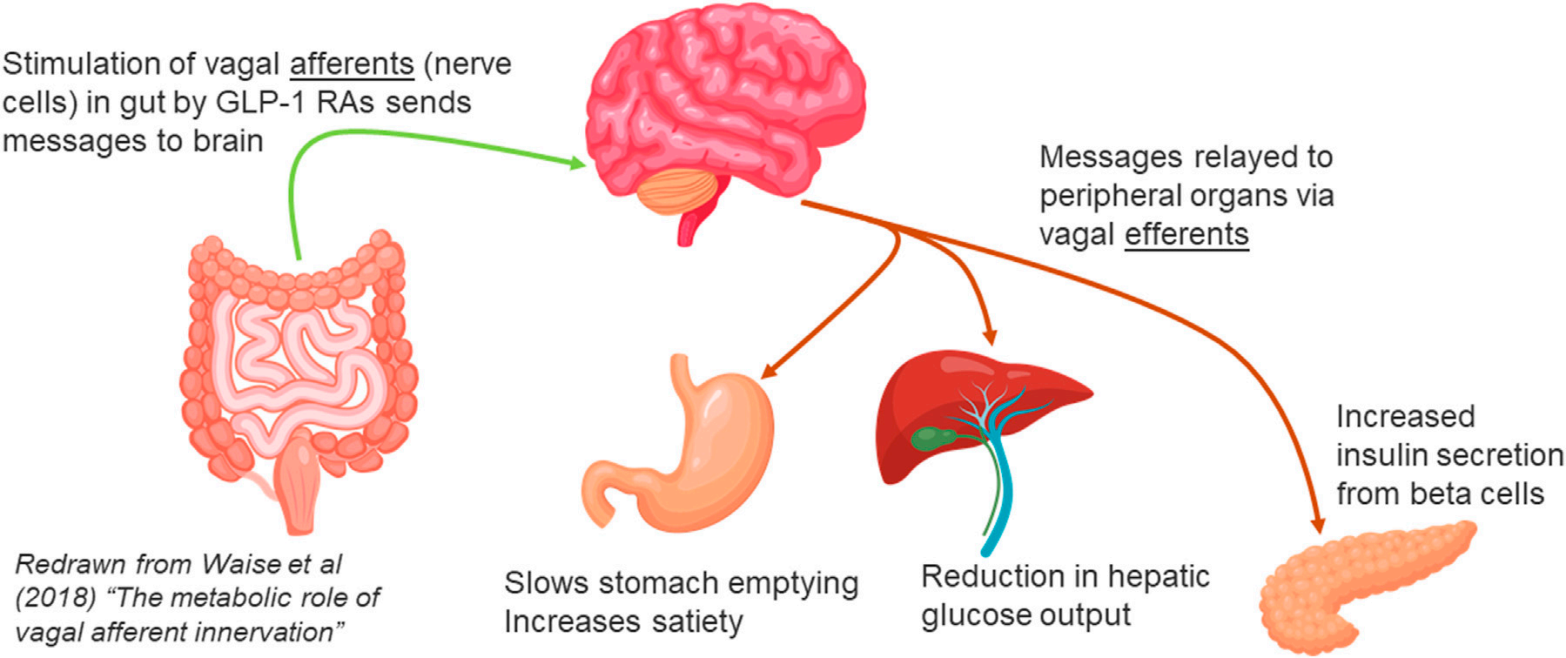
Gut Brain Connection via Vagal Afferent communication.

In normal healthy individuals, ingestion of food stimulates the secretion of glucagon-like peptide-1 (GLP-1) by L cells in the intestine. GLP-1 is rapidly broken down and inactivated by the enzyme dipeptidyl peptidase-4 (DPP4), which is distributed throughout the body, both on the surface of cell membranes, and free in the bloodstream. Before breakdown, however, GLP-1 in intestinal tissues has the opportunity to bind to GLP-1 receptors on the vagal afferent neurons (VAN) projecting into both the lumen and the lamina propria of the intestine. GLP-1 can alter the expression of GLP-1 receptors on the surface of VANs, and binding of GLP-1 to these receptors results in transmission of signals to the brain. The brain then sends messages to organs important in glucose regulation, notably the beta cells in the pancreas, where secretion of insulin is increased, and the liver, to inhibit output of glucose into the bloodstream (Waise et al., 2018; Yang et al., 2017; van Bloemendaal et al., 2014) (Figure 1).

GLP-1 also plays an important role in activating the glucose sensor in the portal vein (Malbert et al., 2021), which can also be accessed by GLP-1 coming from the intestine, before breakdown by DPP4. Since GLP-1 is secreted by intestinal tissue and rapidly degraded in the systemic circulation, its concentration is significantly higher in the intestinal tissues compared to the peripheral circulation.

Because interaction of GLP-1 with receptors in the intestine is sufficient to activate all major responses required to impact both diabetes and obesity, it is anticipated that the administration of GLP-1 receptor agonist (GLP-1 RA) in such a way that they pass across intestinal tissue, should be an ideal method for delivering these peptides. In this paper, three different GLP-1 RAs are studied in animal models to determine the biological responses to apical delivery of GLP-1Ras in the intestine. In the work described here, the pharmaceutical formulation used to achieve oral delivery, Axcess™- comprising a combination of chenodeoxycholate and propyl gallate as absorption enhancers, is one which has already been employed successfully in humans to achieve delivery of insulin in a safe, efficacious and commercially viable manner. (Luzio et al., 2009; New et al., 2022).

GLP-1 RAs recently appearing on the market, in particular liraglutide, semaglutide and tirzepatide (a GIP/GLP-1 RA dual agonist) seek to overcome the problem of the short plasma half-life of native GLP-1 through (i) changing the amino acid sequence of the peptide so that it is no longer broken down by DPP-4, and/or (ii) attaching a long acyl chain to the peptide, thus allowing it to bind to albumin, and avoid rapid elimination via the kidney. These molecules are administered mainly by sub-cutaneous injection, and as such, remain in the peripheral bloodstream for a long period of time (with half-lives of days), but have little access to the intestinal vagal afferents. The proposed mechanism of action of these peptides is that they enter the hind-brain from the peripheral bloodstream, and interact directly with GLP-1 receptors in the brain - possibly the parabrachial nucleus (Kanoski et al., 2016). However, the same molecules can also bind to nociceptive receptors in the brain, and this interaction may be responsible for nausea experienced by a high proportion of patients, which can result in high numbers of patients discontinuing treatment after initiation. In addition, the peptides can interact with receptors in many other parts of the body at high unphysiological levels, which may result in other untoward side effects. Adverse reactions so far reported with these drugs include pancreatic insufficiency (Gier et al., 2012), gallstones (Rehfeld et al., 2018), gastroparesis (Sodhi et al., 2023), non-arteritic anterior ischemic optic neuropathy - NAION (Hathaway et al., 2024) and increase in diabetic retinopathy (Bain et al., 2018). Some or all of these could be by decreased by reducing the quantity of GLP-1 RAs in the outer circulation, and targeting them more to their natural receptors in the intestinal vagal afferents via oral delivery. Given the rate of adoption of GLP-1 RAs in off-label and lifestyle use, there is a significant need for safer versions of GLP-1 RAs, which may result from oral delivery. The studies presented here demonstrate the feasibility and benefits of oral application of GLP-1 RAs, using the Axcess™ vehicle, and the pharmacological rationale for this approach.

## 2 Materials and methods

### 2.1 Materials

Peptides exendin-4 (exenatide) and semaglutide were obtained from Bachem AG, Bubendorf, Switzerland. GLP-1 receptor agonists referred to as G001 and G002 were kindly supplied by Drs Eckhardt Ferdinandi and Claude Larose (formerly of Theratechnologies Inc.). Chenodeoxycholic acid was purchased from ICE Pharma, Reggio Nell’emilia, Italy, propyl gallate was purchased from Panreac Quimica SLU, Barcelona, Spain, and trypsin and chymotrypsin, and corresponding substrates were purchased from SigmaAldrich United Kingdom.

### 2.2 Rat studies

Studies with laboratory rats were conducted in accordance with the standards of the Federal Republic of Germany (similar to AAALAC equivalents) under license numbers SYN01/16, SYN07/20, and SYN01/22, provided by the Regierungspräsidium Tübingen ethics committee.

**FIGURE 2:**
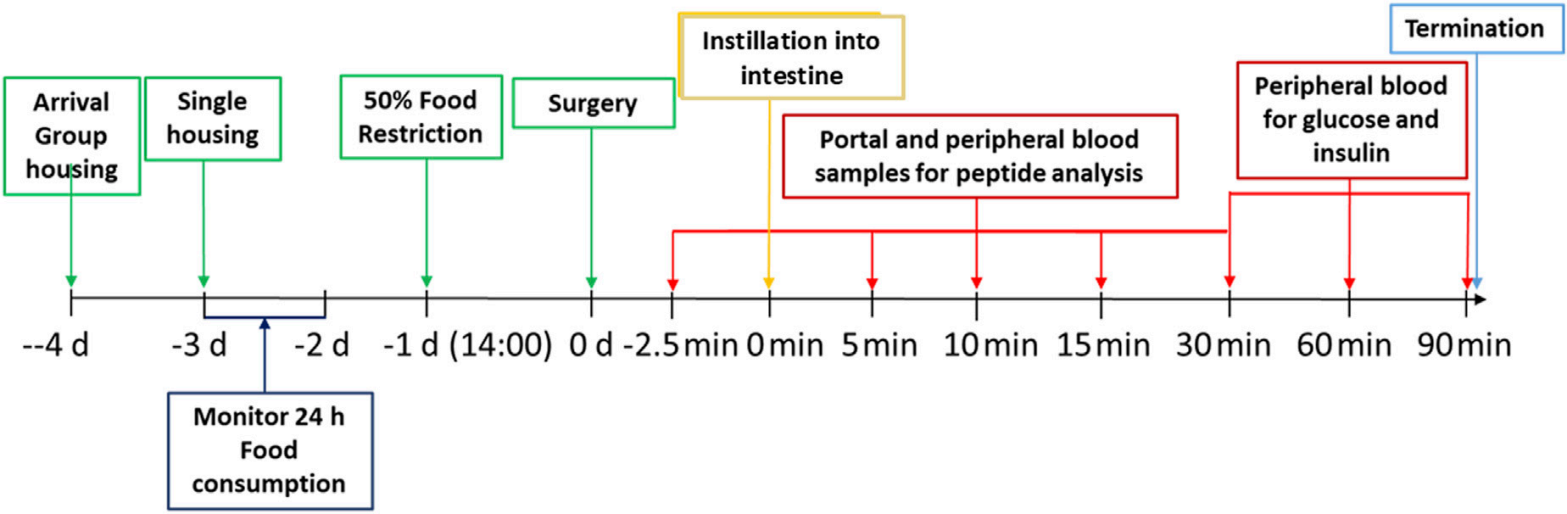
Timeline of the experimental set-up.

Male Sprague-Dawley rats aged 8 weeks were checked for health status on delivery and assigned to cages (2 or 3 animals per cage) within the social groups at delivery and with animals of similar weight in the same cage. Animals were acclimated to the facility for 7 days before transfer to the study room for a further 4 days before the start of the experiment. The rats were randomly allotted to control and treatment groups. Animals were identified by cage tag and corresponding colour tail markings. At day −2 daily 24 h food intake was registered for each cage with 2-3 rats. At 14.00 h on day −1 (i.e., the day prior to the surgery) 50% of the usual food consumed in that interval was offered to the rats. The following day (day 0, from 09:00 to about 15:00 h), rats were anesthetized, using an i.m. injection of 150 μg/kg body weight Medetomidin plus 2 mg/kg bw Midazolam plus 5 μg/kg bw Fentanyl, and subjected to surgery. Intraduodenal injection and all blood sampling was performed in anaesthetized rats. Given ketamine’s pronounced effects on blood glucose, it was specifically excluded from the experimental protocol. The influence of anesthetics and analgesics on glucose and energy metabolism was carefully considered, with particular attention to the potential gut interactions of opioids, which informed the selected dose of fentanyl. The average body weight of the rats at the start of the experiment was 380 g. To groups of five anaesthetized rats, peptides (exenatide or semaglutide) in sterile Dulbecco’s phosphate buffer saline (DPBS) (Biowest, L0615) were administered by intravenous injection via the tail vein. To further groups of five anaesthetized rats, different doses of the peptides formulated in the Axcess vehicle comprising a mixture of 50 mg sodium chenodeoxycholate and propyl gallate (2:1 wt:wt) were dissolved in water and instilled by injection directly into the intestinal lumen of the anaesthetised rats using an insulin syringe with a 30 gauge needle (t = 0 min). Doses of peptides administered are described in the results section. The intravenous route was chosen for administration of the parenteral control since this would ensure greatest reproducibility of entry into the bloodstream, and would match more closely the time course of entry of peptide from the intestine, which is seen to be rapid. Control groups consisting of five animals each were also included, where formulations were tested in which the active was absent (exenatide study), or in which the vehicle contained active, but propyl gallate was omitted from the formulation (semaglutide). At times −2.5, 5, 10, 15 and 30 min post-administration both portal and peripheral blood was collected into EDTA-containing tubes. In addition, peripheral (tail) blood collected at time points 30, 60, and 90 min was assayed for glucose and insulin concentrations. Immediately after the last blood collection at 90 min, the rats were euthanized with carbon dioxide. For an outline of the procedures conducted, see Figure 2 below.

Blood for Exendin-4 and insulin quantification was collected in EDTA containing tubes chilled on ice. To each tube 0.6 TIU/mL aprotinin was added immediately. Portal vein blood samples were taken using an insulin syringe with a 30 gauge needle, peripheral blood samples were taken by puncture of the tail vein. Each blood sample was about 200 μL, resulting in about 100 µL plasma. All blood samples were immediately centrifuged at 1,600 x g for 15 min at 4°C and the resulting plasma subsequently shock frozen in liquid nitrogen. Plasma samples were stored at −80°C until measurement of plasma peptide levels.

Plasma peptide concentrations for each blood compartment and time point in the experiment were measured using commercial ELISA kits (for exendin-4: Creative Diagnostics Exendin-4 ELISA kit (cat. no. DEIABL227) and for rat insulin: Mercodia Ultrasensitive Rat Insulin ELISA (cat. no. 10–1,251-01). For blood glucose measurements one drop of blood from the tail vein was collected and analysed using a handheld blood glucose meter (Accu-Chek Aviva).

All data were entered into Excel spreadsheets and subsequently subjected to relevant statistical analyses (Graph Pad Prism software). Results are presented as mean ± SEM (standard error of the mean) unless otherwise stated. Calculations for biopotency and bioavailability (pharmacological availability) are shown below.

$$Bioavailability=\frac{AUC\;peptide\;enteral\;\text{administration}}{quantity\;of\;peptide\;administered\;enterally}\times \frac{quantity\;of\;peptide\;injected\;i.v.}{AUC\;of\;peptide\;after\;i.v.\;injection}\times 100$$

$$Biopotency=\frac{biological\;activity\;after\;enteral\;\text{administration}}{quantity\;of\;peptide\;administered\;enterally}\times \frac{quantity\;of\;peptide\;injected\;i.v.}{biological\;activity\;after\;i.v.\;injection}\times 100$$

### 2.3 Pig study

A study was conducted in eight domestic pigs in which an indwelling stoma was inserted into the small intestine, so that bile salt-based formulations in capsules could be introduced into the intestine while the animals were conscious. In addition to chenodeoxycholic acid and propyl gallate (2:1 wt:wt), small quantities of glidant (fumed silica) and wetting agent (sodium starch glycolate were included in the encapsulated formulation. The pigs were male, with an average weight of 40 kg. The design and conduct of the study was approved by the ethical committee of Xinjiang Medical School.

Animals were fasted overnight, then administered capsules containing formulated test substances or the same peptides s.c. in free solution, followed by blood sampling at regular intervals from the saphenous vein up to 6 hours after administration, to measure blood glucose levels. The animals were then fed as normal, and the procedure was repeated 2 days later with different test materials. At the end of the study, each animal had received each of the six different treatments selected for comparison.

### 2.4 *In vitro* cell culture

Caco-2 cells (passage #51) were cultured in DMEM (supplemented with 10% FBS) for 3 days on plastic coverslips at the bottom of 1 mL wells in 24-well cluster plates at a density of 1 × 10^6^ cells/mL. 0.5 mL of medium was removed from each well, and replaced with medium containing (i) FITC-insulin alone (concentration 100 μL/mL), (ii) FITC-insulin with sodium chenodeoxycholate (1 mg/mL) or (iii) FITC-insulin with sodium chenodeoxycholate (2 mg/mL). The cells were incubated at 37 °C in 5% CO_2_ for half an hour. The supernatant was then removed, replaced with 0.5 mL of 4% paraformaldehyde solution, and incubated at room temperature for 15 min. The paraformaldehyde was then removed, the wells were washed three times with phosphate-buffered saline, and the coverslips then treated with mounting medium and viewed under a confocal microscope.

### 2.5 Enzyme inhibition studies

Trypsin, from bovine pancreas, was dissolved in Hanks Balanced Salt Solution (HBSS) at a concentration of 0.02 mg/mL. The substrate Z-L-arginine 4-methyl-7coumarinylamide was dissolved in HBSS at a concentration of 0.01 mg/mL, and sodium chenodeoxycholate was dissolved at a range of concentrations starting at 100 mg/mL. The assay was conducted in the wells of 96-well black plastic microplates, in which 20 μL of enzyme solution and 60 μL of bile salt were mixed together, and then 20 μL of substrate was added, with mixing, at the start of the reaction, which proceeded at room temperature for up to 30 min, with measurement at various intermediate time points.

In a second study, the procedure above was repeated but with chymotrypsin as the protease (instead of trypsin) and with glutaryl-L-phenylalanine 4-methyl-7-coumarinylamide as the substrate, and a dose response curve with chenodeoxycholate at different concentrations was constructed.

## 3 Results

### 3.1 Uptake of exendin-4 in rats

In order for peptides to be delivered by the Axcess formulation, the combination of API and excipients needs to pass through the stomach in an enteric-coated capsule, to prevent dilution or damage to the formulation, by acid conditions in the stomach. Unfortunately, reliable administration of capsules to the rat in such a way that they can pass through the stomach in a timely fashion is not possible. Consequently, in this study, rats were anaesthetised, and the formulation instilled directly into the intestine after the powder is converted to an aqueous solution.

**FIGURE 3:**
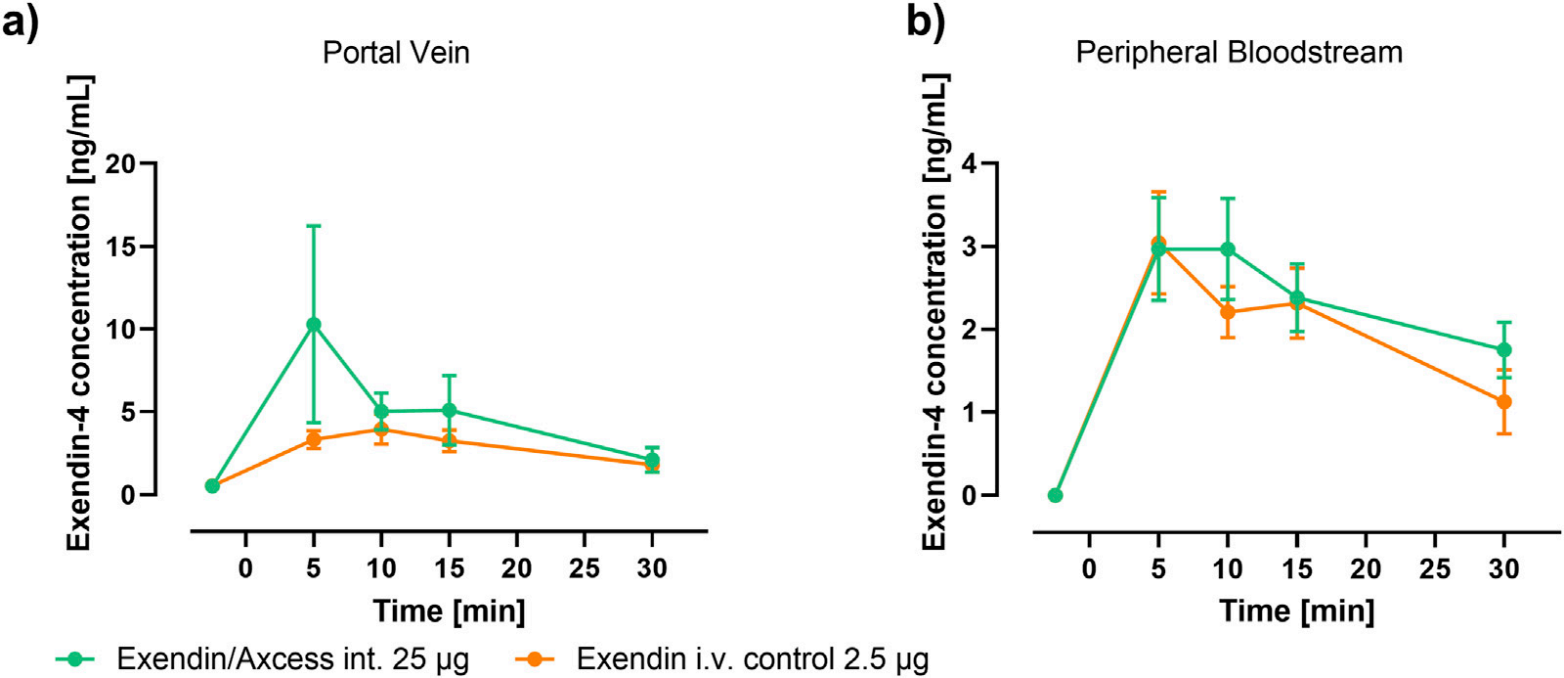
Time-course of Plasma Exendin-4 following application of Axcess-formulated exendin-4 (green) and control exendin-4 (orange) in rats sampled from **(A)** the portal vein and **(B)** periphery (tail). Data are represented as means ± standard errors (n = 5). Int: application to the intestinal lumen; i.v.: intravenous application.

Levels of exendin-4 appearing in the plasma of anaesthetised rats after administration either via the intestine in the Axcess formulation (25 μg per animal), or i.v. via the tail vein as a free solution (2.5 μg per animal) in phosphate buffered saline, are shown in Figure 3.

Because the half-life of this small peptide is short, levels reach a peak, then fall off rapidly with time. The highest levels are seen in the portal vein, after intestinal administration of the formulated peptide, while in the peripheral bloodstream similar levels are observed for both routes of administration. Blood concentrations achieved with a control formulation in which propyl gallate was omitted were only slightly above background, demonstrating the importance of the combination of components employed in the Axcess delivery vehicle, to achieve optimal activity. Values of C_max_ and T_max_ are shown in Table 1 below.

**TABLE 1:** Pharmacokinetics of exendin-4 in portal and peripheral blood after administration via the intestine, or by intravelous injection. Values are ± standard errors of the mean.

	25 μg Exendin-4 via intestine in axcess		25 μg Exendin-4 via intestine in axcess control		2.5 μg Exendin-4 i.v. in PBS	
	Portal vein	Periphery	Portal vein	Periphery	Portal vein	Periphery
Cmax (ng/mL)	10.3 ± 5.9	3.0 ± 0.6	2.6 ± 1.0	0.7 ± 0.2	3.9 ± 0.9	3.0 ± 0.6
Tmax (minutes)	5	5	5	10	10	10
AUC (0–30) min.ng/mL	144.7 ± 34.9	60.9 ± 9.5	36.2 ± 12.4	15.8 ± 4.1	82.5 ± 7.6	66.7 ± 8.2

Taking differences in quantities of peptide administered via the intestine compared with i.v. application (a 10:1 ratio wt:wt intestine vs. i.v.) the bioavailability of the exendin-4 administered via the intestinal route is 19% for delivery to the portal vein, and 9% in the peripheral bloodstream. The bioavailability in the portal vein is important, since there are GLP-1 receptors in both the portal vein, and on the membranes of vagal afferent nerve cell in the intestine, which will be accessed by the peptide before it reaches the outer circulation.

**FIGURE 4:**
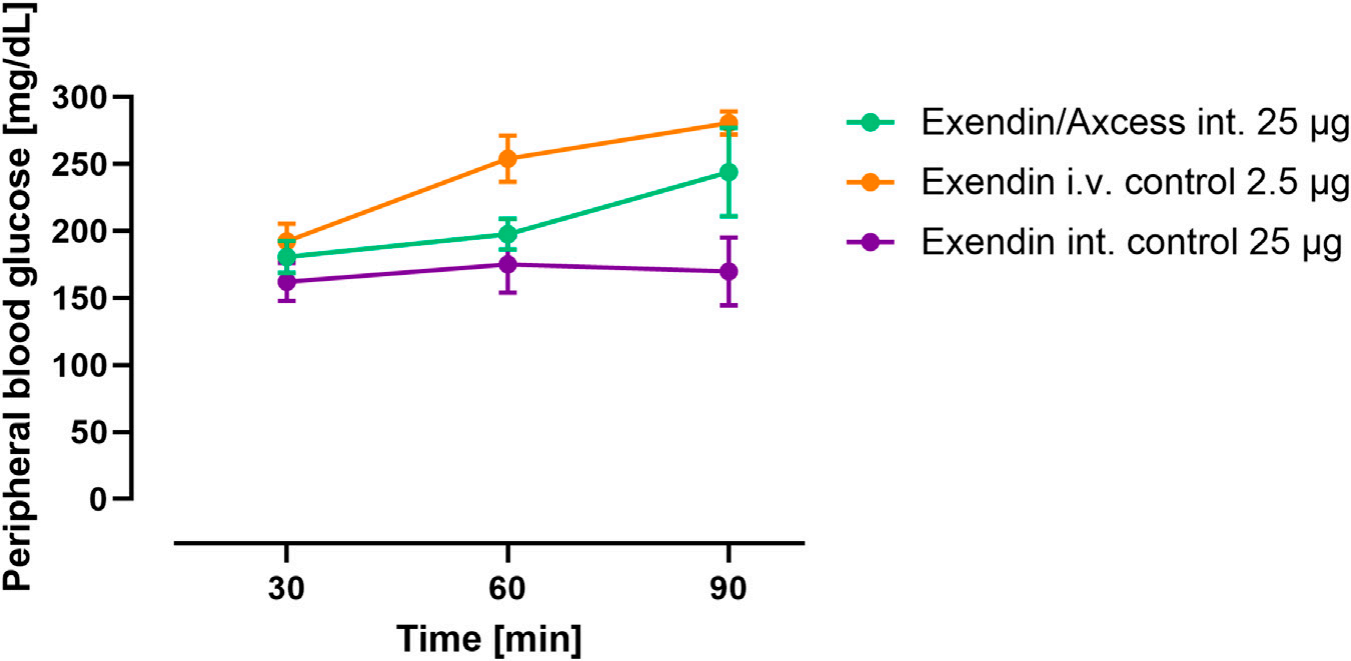
Pharmacodynamics of intestinal exendin-4 (green: Axcess formulated exendin-4, purple: control formulated exendin-4) compared with intravenous administration (orange): changes in blood glucose levels. Data are represented as means ± standard errors (n = 5). Int, application to the intestinal lumen; i.v., intravenous application.

At the same time as the above measurements were taken, blood glucose concentrations were determined, in order to seek evidence of a biological effect due to the exendin-4 administered. As can be seen in Figure 4, exendin-4 given i.v. brought about an increase in blood glucose over a 90 minute period. While it is known that GLP-1 receptor agonists bring about falls in glucose under normal circumstance, due to stimulation of insulin secretion and inhibition of hepatic glucose output, the response to high doses of exendin-4 in the anaesthetised rat is to increase blood glucose levels, and this has previously been reported elsewhere by Perez Tilve and co-workers (Perez-Tilve et al., 2010), with the effect being attributed to activation of the sympathetic nervous system. Another aspect that impacts these results is that fasting appears to alter GLP-1 response. The anorexic effects of GLP-1 analogs appear to require adequate glucose supply (Sandoval and Sisley, 2015), which is absent in fasted animals. Thus, studies in fasted animals prepared for surgery will potentially provide data that are paradoxical *versus* the fed setting. Interestingly, the glucose levels in rats receiving exendin-4 via the intestine also increased and comparison of the AUCs, taking into account the difference in quantity of peptide administered suggests a relative biopotency of 7%. Biopotency in this context means the pharmacodynamic effect of a given amount via the intestinal route vs the same dose i.v.

**FIGURE 5:**
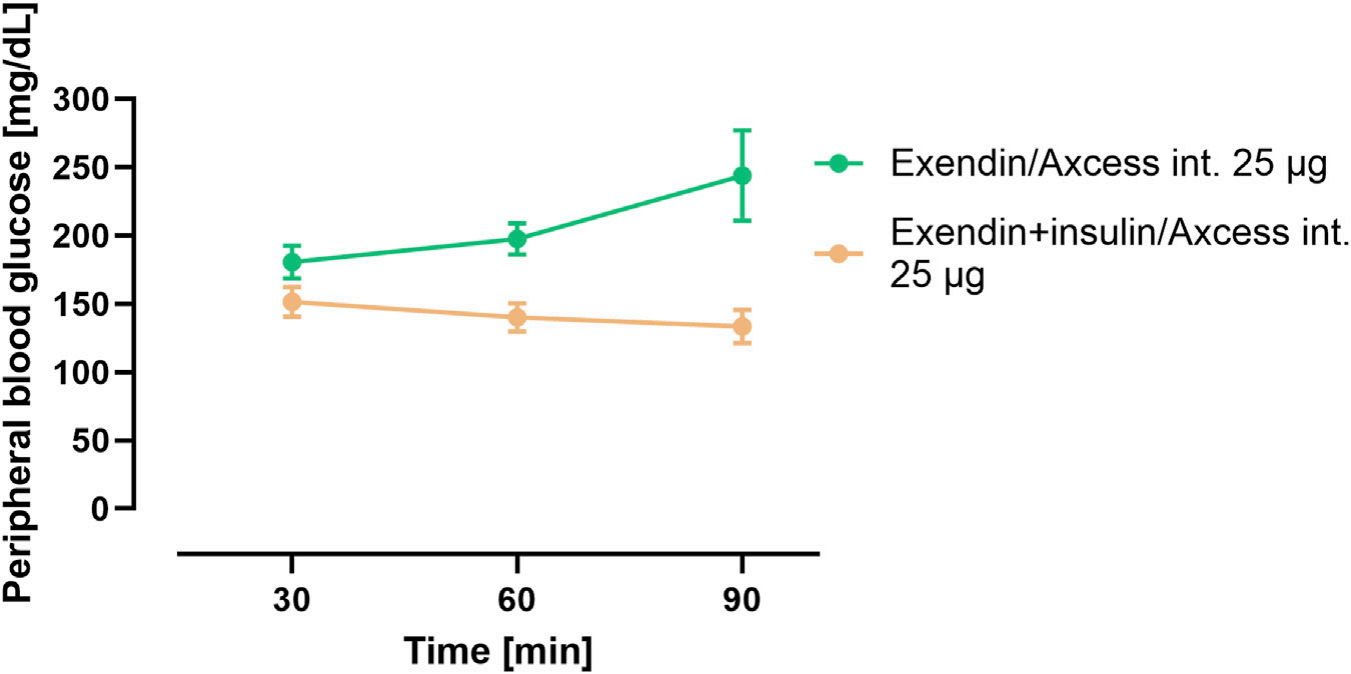
Blood glucose concentration after administration of exendin-4 in the Axxess formulation alone (green), or co-administration of insulin and exendin-4 in combination (brown). Data are represented as means ± standard errors (n = 5). Int, application to the intestinal lumen; i.v., intravenous application.

In a third group in this study, the same quantity of exendin-4 was administered via the intestine co-formulated in the Axcess formulation with recombinant human insulin. In this combination insulin appears to reduce blood glucose relative to exendin-4 administered alone (Figure 5). These data confirm that insulin is also delivered in a functionally active form by this formulation.

### 3.2 Administration of semaglutide to rats via the intestine

**FIGURE 6:**
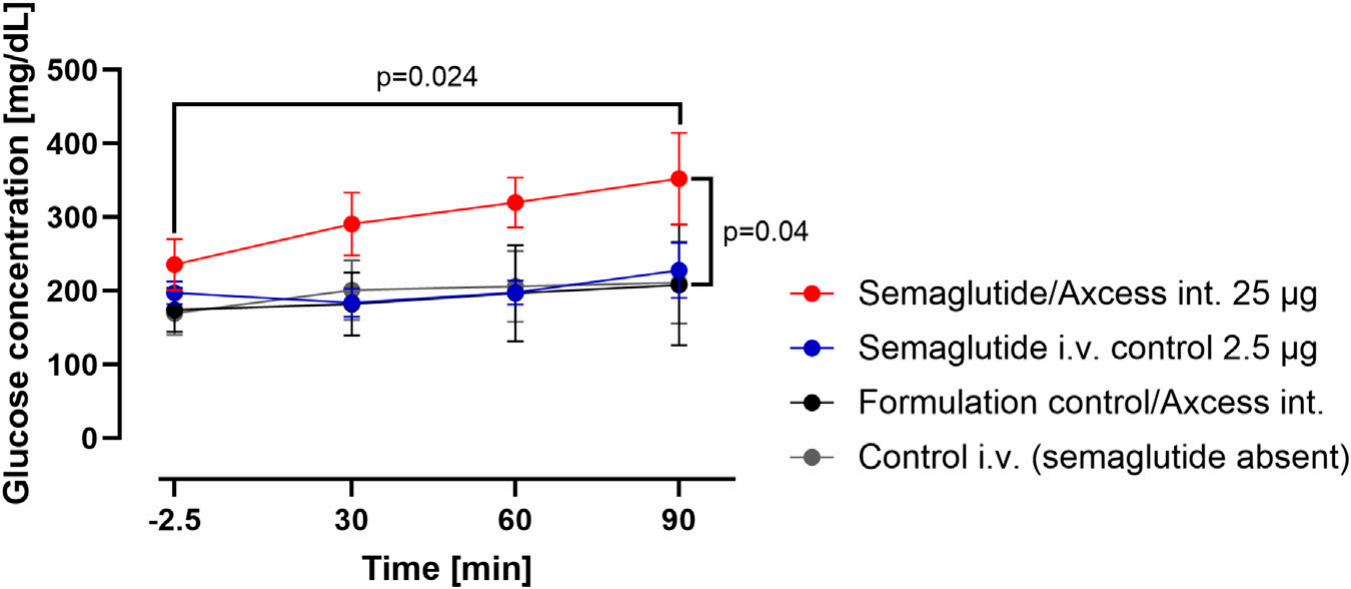
Change in glucose levels in peripheral blood after administration of semaglutide either in the Axcess formulation via the intestine (red), or in free solution after i.v. injection (blue). Formulation control was the same formulation containing a non-active peptide injected via intestine (black) or i.v. (gray). Data are represented as means ± standard errors (n = 5). Int, application to the intestinal lumen; i.v., intravenous application.

A study was conducted in an identical manner to that described above, except that semaglutide (intravenously administrated in a dose of 2.5 µg) i.v. or administered into the intestinal lumen in a dose of 25 µg was employed instead of exendin-4. Glucose levels in blood after intestinal administration of semaglutide exceed those achieved after intravenous administration (Figure 6). This appears to be consistent with the observations made for exendin-4 under the same circumstances.

**FIGURE 7:**
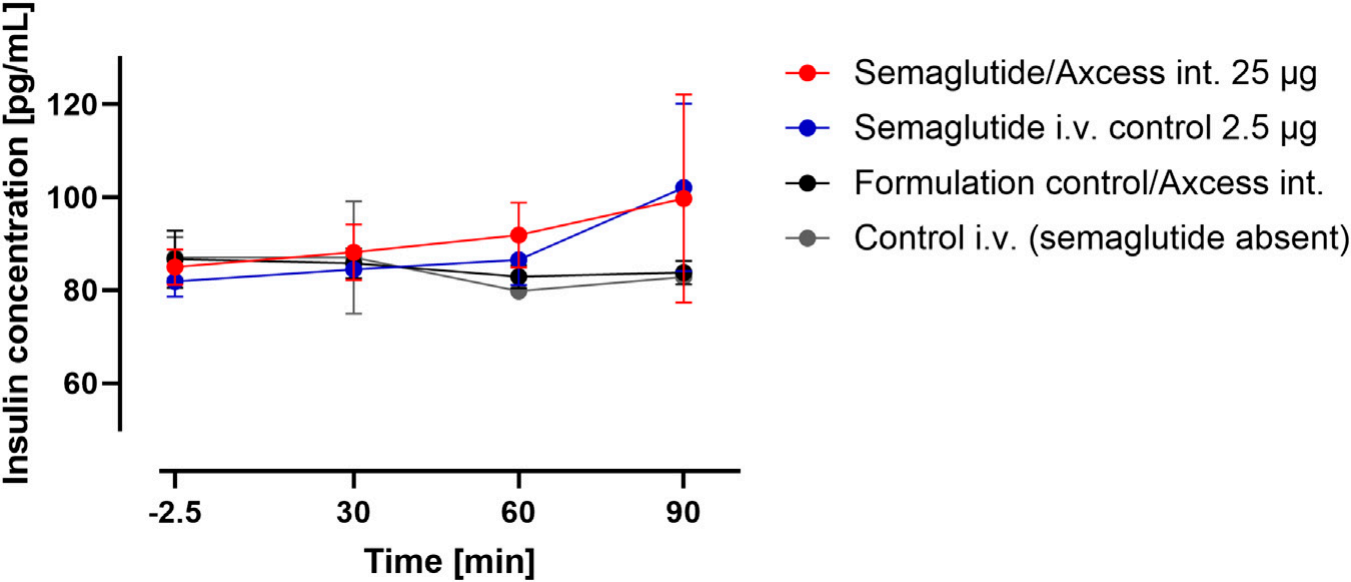
Insulin levels in peripheral blood after administration of semaglutide either in the Axcess formulation via the intestine (red), or in free solution i.v (blue). Formulation control was the same formulation containing a non-active peptide injected via intestine (black) or i.v. (gray). Data are represented as means ± standard errors (n = 5). Int, application to the intestinal lumen; i.v., intravenous application.

Plasma insulin levels sampled from the tail tended to rise from baseline about 60 min after application of semaglutide either i.v. or via the intestinal lumen. Insulin levels remained stable following application of vehicle by either the i.v. or intestinal routes. These data suggest that semaglutide administered via the intestine is able to elicit a normal insulin response (Figure 7).

**FIGURE 8:**
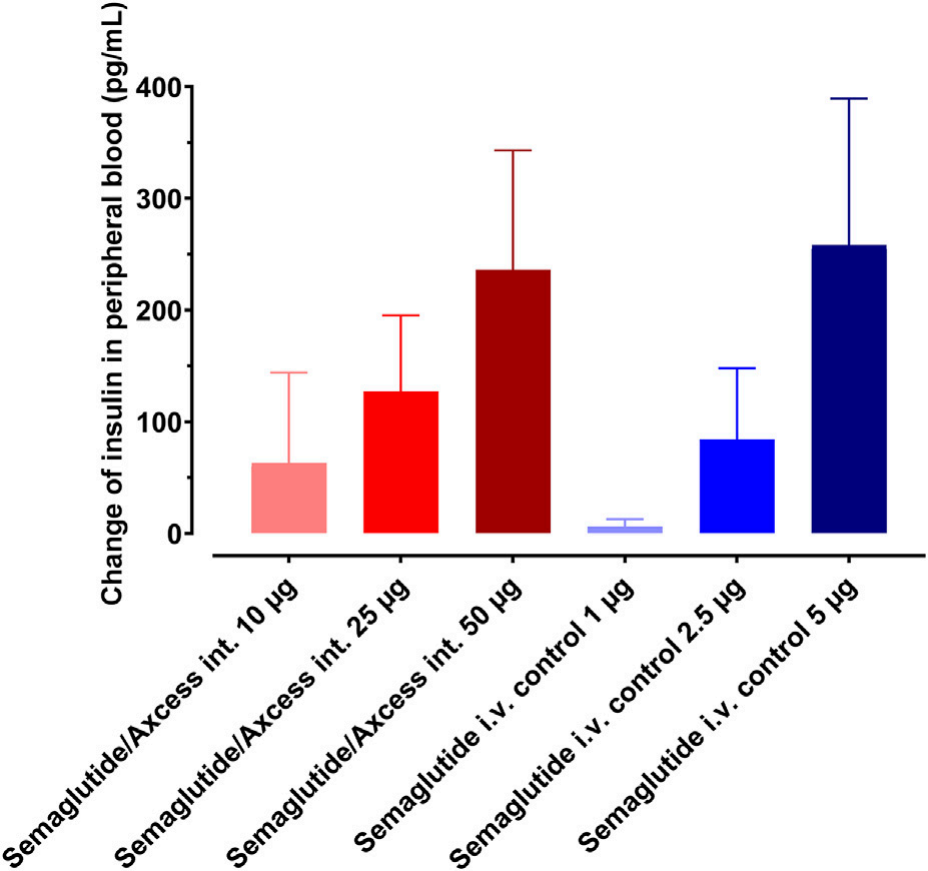
Dose response to semaglutide administered by Axcess formulation via the intestine (red tones), or as a free solution i.v. (blue tones). Response is determined by insulin secretion elicited by semaglutide. Different shades represent different concentrations. Data are represented as means ± standard errors (n = 5).

The study was repeated using the same rat model to compare responses to 10, 25 and 50 µg semaglutide applied to the intestinal surface *versus* 10-fold lower doses applied via the i.v. route. Dose-response relationships in terms of insulin levels in tail plasma are apparent for both routes of application (Figure 8). The intestinal application trends to be relatively more potent at lower doses, while at 50 μg, the response to the intestinal application is similar to 5 µg i.v. suggesting that above a certain threshold, the i.v. material becomes more available to intestinal and pancreatic receptors while at lower doses, the intestinal route provides more direct exposure to the relevant receptor locations. Tukey pair-wise comparison for both routes indicated a significant difference between highest and lowest doses (*p* = 0.046).

A regression analysis demonstrated linearity in the dose relationship, with *p* < 0.05 in each case. On the basis of the differences in the gradients, an average relative biopotency of 14.8% could be inferred for intestinal administration over the full dose range, albeit with this likely to be higher if assessing only lower doses.

### 3.3 Activity of GLP-1 analogues in pigs

In a separate experiment, intestinal delivery was studied of two GLP-1 analogues where modifications were made with the aim of increasing their circulating half-life by several hours relative to the native molecule. In one variant, G001, the molecule was identical to native GLP-1, except that N-terminal amino group was conjugated with salicylic acid. In the second, lipidated, analogue, G002, the N-terminal was conjugated with trans-3-hexenoic acid, and in position 14, leucine was replaced by octyl glycine. In addition, glutamate and alanine in positions 21 and 24 respectively were reversed, and a glycine residue at position 29 was replaced by asparagine. The longevity of action of G001 and G002 differed markedly, their half-lives in rats being 25 min and ∼12 h respectively (unpublished data). The sequences are shown below:

G001: Sal-HAEGTFTSDVSSYLEGQAAKEFIAWLVKGR-NH_2_

G002: t3h-HAEGTFTSDVSSYZEGQAAKAFIEWLVKN-NH_2_

Modification of the N-terminal histidine with acyl or benzoyl derivatives confers partial resistance to attack by DPP4, while introduction of a straight-chain alkyl group in position 14 of G002 helps to slow down elimination by the kidney by encouraging association with lipidic pockets in large proteins such as albumin or lipoproteins. The same result may be achieved by redistribution of charged residues to align along one side of the C-terminal alpha helix, with predominantly hydrophobic amino acids on the opposite side (Peri K. et al., 2004).

The juvenile pig was chosen as a large animal model because the pig has a small intestine closer in physiology to humans than any other common animal models. The pig is also similar to humans in weight, length of intestine, and intestinal blood flow. While capsules can be administered by mouth to pigs, it was decided to introduce the capsule directly into the jejunum, since the gastric transit in the pig can be variable and of long duration, the pig stomach is more fermenting than that of a human, and the pH of the stomach is high, to the extent that enteric coating of the capsule may not give adequate protection. Consequently, the animals were surgically prepared in advance of the experiment to allow direct access to the lumen of the gut from the outside via an in-dwelling stoma.

Capsules (non-enteric coated), containing each of the GLP-1 analogues combined with the Axcess delivery formulation (66 mg chenodeoxycholic acid and 33 mg of propyl gallate) were introduced into the intestine after fasting overnight, and blood samples were taken via the saphenous vein for 6 hours thereafter, prior to feeding. A placebo capsule, containing excipients but no peptide, was also administered in the same way, and peptide administered as a free solution by injection was also tested. The study was conducted in a cross-over design, with a 2-day wash-out period between each dose.

**FIGURE 9:**
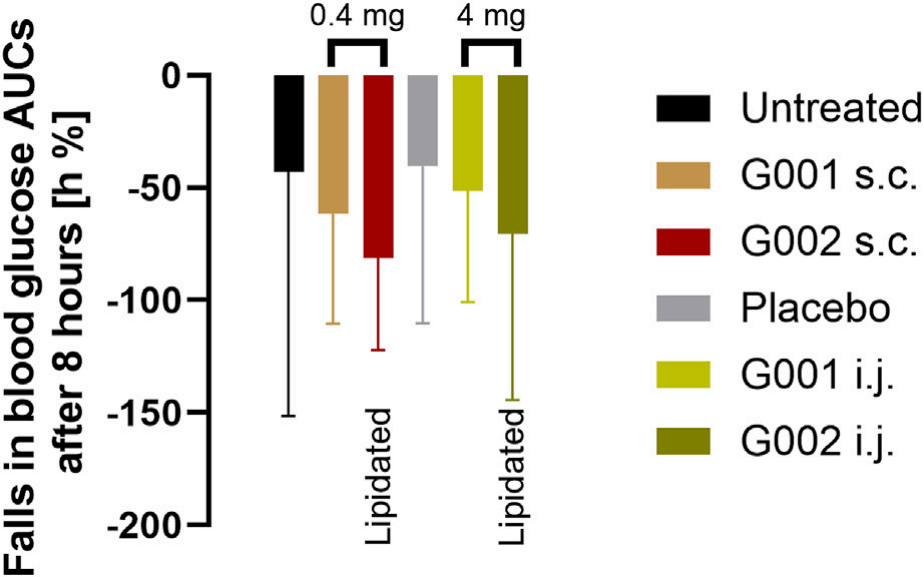
AUC of glucose response after administration of peptides, either 0.4 mg as solution s.c. (brown and dark red) or 4 mg in the Axcess formulation via the intestine (intra jejunal, i.j.) (light and dark green).

In this format, blood glucose concentrations decreased over time because animals were fasted overnight, and feeding deferred until the final blood sample was taken. The model, therefore, measures effects of the peptides on pancreatic insulin secretion and consequent additional blood glucose reduction. Treated animals consistently recorded lower blood glucose than vehicle treated animals (Figure 9). When injected s.c. in free solution, G002 had a stronger effect than G001. Although statistical significance was not achieved, a similar trend was seen for the peptides administered via the intestine. Taking into account the difference in dose administered via the two routes (a 10:1 ratio wt:wt intestinal:parenteral) a biopotency of ∼7% is calculated for G002.

### 3.4 Mechanisms of action of the Axcess formulation

The success of the Axcess formulation in delivering peptides via the intestine resides in two key properties of the excipients. One is the ability of the excipients to inhibit the activity of proteases in the intestine. The other is the action of the excipients in stimulating enterocytes to take up material from the extra-cellular fluid by a process of vacuolation.

**FIGURE 10:**
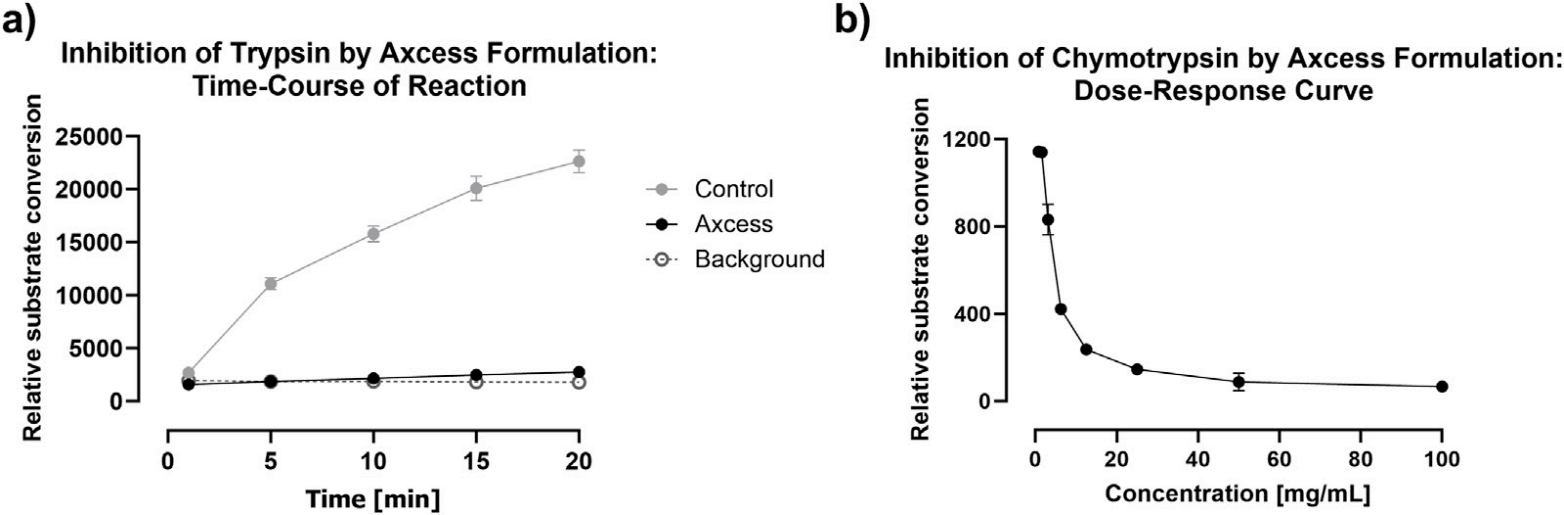
Inhibition of intestinal proteases by the Axcess formulation mixture chenodeoxycholic acid and propyl gallate (2:1 wt:wt). **(A)** inhibition of trypsin after Axcess formulation (black circles), control (grey circles) or background (open circles), **(B)** inhibition of chymotrypsin by Axcess formulation.

With regard to intestinal proteases, the most important examples in the gut are trypsin and chymotrypsin, which are secreted by the pancreas, and are present in significant quantities throughout the gut lumen. These have specificities for cationic residues (arginine and lysine) and aromatic residues respectively, so that most therapeutic proteins are vulnerable to attack by one or both of these enzymes. Figure 10 demonstrates the ability of Axcess excipients to inhibit the activity of both trypsin and chymotrypsin, using fluorogenic substrates to monitor the degradation of these peptides to release fluorescent breakdown products. Robust proteolytic activity is observed over a 20 minute period in the absence of Axcess excipients, which is completely ablated in their presence (Figure 10A).

The excipients only exert their activity at high concentration, which means that, while they are able to prevent proteolysis as soon as they are released from the capsule, peristaltic action and dilution with luminal fluid result in reduction of their inhibitory activity after a certain time window–probably no longer than 30 minutes. This means that protease inhibition acts to protect the API during it passage across the enterocyte, but concerns about long-term interference with digestive process in the gut are absent, because of the transient nature of the inhibitory activity deployed. The relationship between proteolytic activity and excipient concentration is shown for chymotrypsin in Figure 10B.

**FIGURE 11:**
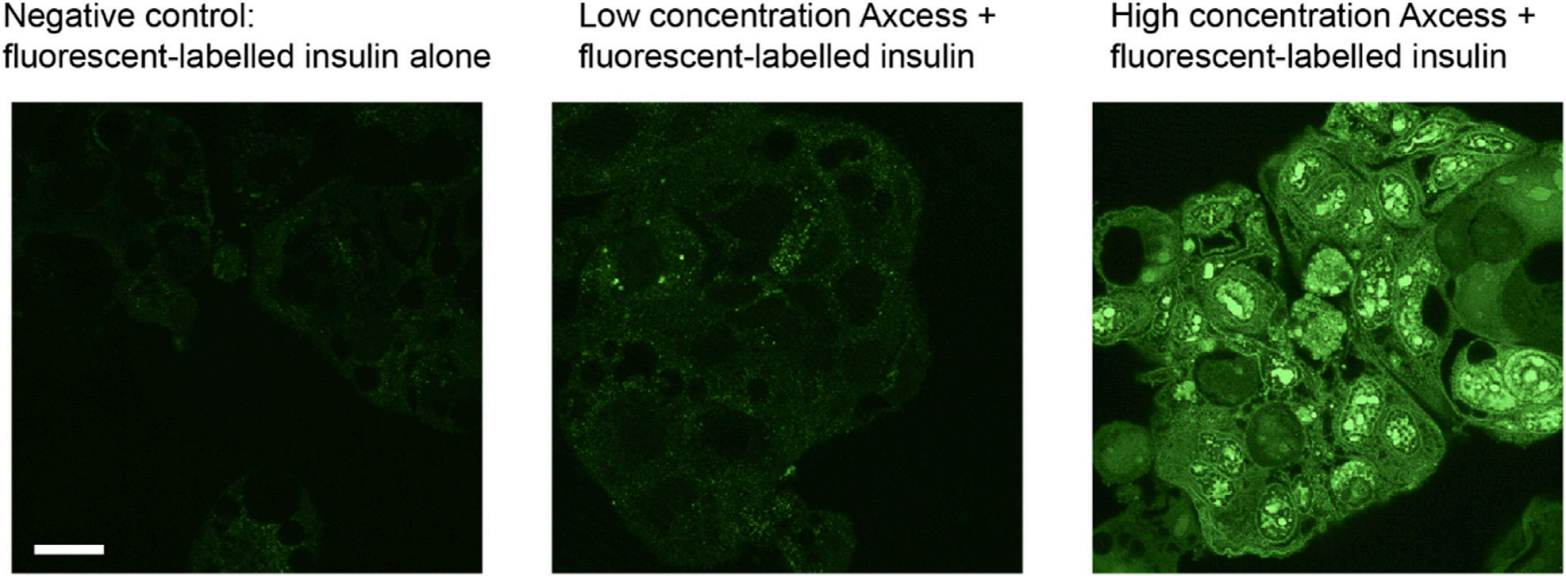
Uptake of fluorescein-labelled insulin into CaCo2 intestinal cells in culture in the presence of components of the Axcess formulation (1 mg/mL) viewed by confocal microscopy. The scale bar represents 20 μm.

Stimulation of uptake of peptides by intestinal cells is shown clearly in *vitro* experiments using a Caco-2 cell model in the presence and absence of the Axcess excipients. Cells cultured in 12-well plates were incubated for 30 min with fluorescein-labelled human insulin, then washed to remove unbound material, and observed using fluorescence microscopy. In each of the panels shown in Figure 11 the same number of cells is present, but only cells exposed to high levels of excipients exhibit fluorescence, since only these cells have taken up significant quantities of insulin (the fluorescein is not cleaved from insulin under these conditions).

The labelled insulin appears inside the cells as punctate foci, probably within early endosomes. Experiments with inhibitors of different cell uptake pathways indicate that internalisation of peptides takes place through involvement of clathrin-coated vesicles. The process is not limited to insulin, since other molecules such as bovine serum albumin and hGH are taken up in the same way. Uptake by vacuolation, and transport across the cell within vesicles, followed by exocytosis through the basal membrane, is a process taking place naturally for the absorption of nutrients into the rest of the body. The Axcess delivery system is thus harnessing this natural mechanism of uptake to effect transcellular transport of intact proteins across the gut wall.

## 4 Discussion

In 2019, an oral form of semaglutide was approved for treatment of diabetes, and subsequently obesity, namely, Rybelsus. This uses the Eligen delivery vehicle, employing Salcaprozate Sodium (SNAC–a capryl benzyl amide), as an absorption enhancer. The site of absorption has been reported to be the stomach (Buckley et al., 2018), with only a small quantity of the peptide reaching the small intestine. Consequently, this route of delivery misses the opportunity to interact with the majority of GLP-1 receptors in the intestine, so the vagal afferents mechanism of action is unlikely to make a large contribution to its biological activity. The mode of action can be attributed to the entry of the drug from peripheral blood into the brain, in a similar way to its injected counterparts.

Although there are GLP-1 receptors in the stomach wall, these appear to be mechanoreceptors, and control local motility of the gastric tissue, rather than being involved in direct messaging to the brain (Williams et al., 2016). Maybe for this reason, the biopotency of semaglutide in Rybelsus is low, having been calculated to be 0.4% in humans (Abramson et al., 2019) with a bioavailability of 0.8% (Overgaard et al., 2021), which necessitates extremely large amounts of semaglutide to be incorporated into the tablet to achieve a clinical effect.

The Axcess™ formulation was originally developed by Diabetology in 2003, and recent improvements have increased efficacy further, increasing the scope of the associated intellectual property, and providing cover out to 2040 and beyond. The original Diabetology Axcess™ formulation (chenodeoxycholate and propyl gallate in a 2:1 ratio wt:wt) has been applied in dogs, using a GLP-1 RA which has been modified to resist not only DPP4 attack, but also breakdown by intestinal proteases (Pechenov et al., 2021). This preparation provided a bioavailability of ∼6% which is consistent with our work reported here. Although no relative biopotency figures have been reported by Pechenov et al., the study showed that the Axcess formulation was superior in terms of bioavailability to other common absorption enhancers such as capric acid and tetradecyl maltoside, emphasising the importance of the chenodeoxycholate and propyl gallate mixture developed by Diabetology.

The work reported by others in dogs, as well as our own results reported here in pigs and rats, show that the Axcess™ formulation is able to deliver GLP-1 receptor agonists in effective amounts, even at low doses, to the intestinal mucosa. More importantly, the formulations exert potent biological action at this site. This is significant because it is the natural site of action of the GLP1-RAs and most relevant to their uses in diabetes and appetite control. The high potency of application to the gut mucosa will be because the peptides stimulate the vagal afferents directly in the intestine. In contrast, s.c. application requires sufficient dose to occupy carrier protein sites in plasma and simultaneously exposes non-target tissues like the hindbrain in supra-physiological concentrations. The combination of high initial levels after injection and brain exposure is considered the likely cause of side effects such as nausea and vomiting. In contrast, oral application is compatible with daily, timed use which is more likely to achieve normal physiological effects. It is clear that although GLP1-RAs will be useful in treating diabetes, and probably Alzheimer’s disease, the vast majority of its use will be in appetite control and associated weight management. This is an application that has a completely different magnitude of use rates and thus, overall consequences for long-term side effects. Given that appetite and weight control will be key targets for an oral formulation, it is fortuitous that it is the stimulation of vagal afferents that has a direct impact on satiety effects of GLP1-RAs (Cork, 2018). This suggests that oral delivery and the Axcess type formulations will be a more specific and effective treatment not only for diabetes but for obesity as well.

## Data Availability

The original contributions presented in the study are included in the article/supplementary material, further inquiries can be directed to the corresponding author.
